# Bridging international borders through global health diplomacy: A comprehensive bibliometric analysis of the state of play and leads for advancing this domain

**DOI:** 10.34172/hpp.025.44650

**Published:** 2025-11-04

**Authors:** Gaurav Chanderprakash Mittal, Vijay Kumar Chattu, Rahul Clare, Prakash Narayanan, Cherian Varghese

**Affiliations:** ^1^Center for Evidence-based Diplomacy, Global Health Research and Innovations Canada Inc. (GHRIC), Toronto, ON, Canada; ^2^ReSTORE Lab, Department of Occupational Science and Occupational Therapy, Temerty Faculty of Medicine, University of Toronto, Toronto, ON M5G 1V7, Canada; ^3^Central Asian Regional Center for Planetary Health (CARCPH), Semey Medical University, Semey, 071400, Kazakhstan; ^4^Department of Public Health, Health Administration & Information and Health Sciences, College of Health Sciences, Tennessee State University, Nashville, TN 37203, USA; ^5^Centre for Digital Health, Applied Research, and Technology, Prasanna School of Public Health, Manipal Academy of Higher Education (MAHE). Manipal – 576 104, India; ^6^Department of Global Public Health Policy & Governance, Prasanna School of Public Health, Manipal Academy of Higher Education (MAHE). Manipal – 576 104, India; ^7^Prasanna School of Public Health, Manipal Academy of Higher Education (MAHE). Manipal – 576 104, India

**Keywords:** Bibliometrics, Climate, Digital health, Diplomacy, Global health, International cooperation, Low- and middle-income countries (LMICs), Noncommunicable diseases (NCDs), Sustainable development, Vaccines

## Abstract

**Background:**

Global health diplomacy (GHD) is an emerging intersection of health and international relations, particularly in transnational health challenges. Though growingly important, especially in the current global health scenario, this study aimed to perform a bibliometric analysis of GHD to identify emerging themes, leading contributors, research gaps for further studies and policy directions.

**Methods:**

A bibliometric analysis was done on SCOPUS, and a return of 242 articles published between 2007 and 2024 contained the keyword "global health diplomacy." The data was analyzed using Biblioshiny and then exported to Microsoft Excel for thematic coding. Key indicators included publication trends, co-authorship networks, and keyword co-occurrences to establish key trends and gaps.

**Results:**

A growing body of research observed an annual growth rate of 7.65% [95% CI]. North American and European countries led the research, especially the United States, Canada, and the United Kingdom. The dominant themes included vaccine diplomacy, global health, Artificial Intelligence-Machine Learning and digital health, governance, and international cooperation. However, there were significant gaps, including underrepresentation from low-and middle-income countries (LMICs), limited focus on noncommunicable diseases (NCDs) including mental health, and neglected climate-health intersections.

**Conclusion:**

This study highlights the fast growth and changing nature of GHD research while indicating some key gaps that deserve further research. Strengthening contributions of LMICs, expanding thematic focus to NCDs and environmental health, and fostering interdisciplinary approaches are crucial for advancing the field. The findings are highly relevant for policy and research purposes and will push forward an impactful GHD for global health challenges.

## Introduction

###  The convergence of health diplomacy, security, and development

 Health diplomacy can play at different levels, global, transnational, regional and even within countries, it now represents a potent tool at the nexus of global health^1^ international relation, and global development.^2,3^ It encompasses efforts to strike negotiations and form alliances to tackle transboundary health issues while realizing broad geopolitical and economic objectives.^4^ The concept of “Health diplomacy” was introduced as early as 1978 by Peter Bourne,^5^ special assistant to the president on health issues during the Carter administration. He argued that “the role of health and medicine as a means for bettering international relations has not been fully explored by the United States. Certain humanitarian issues, especially health, can be the basis for establishing a dialogue and bridging diplomatic barriers because they transcend traditional and more volatile and emotional concerns”.^4^ This concept emerged and developed over the past few decades, and the terminology of global health diplomacy (GHD) is now mainstream among policymakers and researchers because of the pioneering research in this area.^6-9^ One hundred-nineteen articles of the 172 articles on the topic ‘global health diplomacy’ published in peer-reviewed scientific PubMed^10^ journals appeared just in the last decade (2014–2024), showing the increasing importance of the field in shaping global health.

 One of the most striking themes within GHD is vaccine diplomacy,^11^ i.e., vaccines are dedicated instruments to close gaps, ensure health equity and build bilateral and multilateral relations, particularly seen during the COVID-19 pandemic.^12^ This top-down strategy of vaccine diplomacy has a long and documented history apart from the most recent pandemic, from the success of smallpox elimination by global cooperation^13^ to the eradication of polio in many countries in Southeast Asia^14^ and ongoing efforts across sub-Saharan Africa.^15^ In the current landscape of fast-moving healthcare systems, efforts at health diplomacy have now broadened–not only to encompass health security, economic development, and so on but also to strengthen multilateralised frameworks^16^ such as the Group of 20 (G20),^17^ Group of 7 (G7),^18^ The Network: TUFH^19^ etc. These actions highlight the tight relationship of health systems, trade policies, and global development aspirations.^7,20^ For instance, the G20 (G20 2025 Health Working Group) recently highlighted the importance of health resilience in promoting economic stability, connecting pandemic preparedness to green growth, and calling for equitable access to health technologies as a shared global endeavour.^21^

###  Public health challenges and global burden of disparities

**Figure 1:**
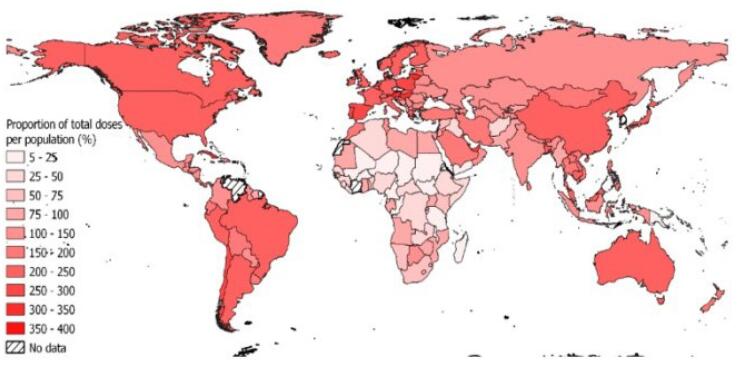
Total vaccine doses delivered as a population percentage per country. (source: ourworldindata.org/covid-vaccinations)^25^

 GHD fundamentally involves health security, that is, the protection of populations from health threats that cut across borders.^22^ Recent crises illustrate the devastating impact of insufficient health security. The COVID-19 pandemic, for example, caused global economic losses and disproportionately affected low-and middle-income countries (LMICs).^23^ Globally, 67 countries received less than 100%, and 35 countries received less than 50% of the vaccine doses as a proportion of their total population. Among these 35 countries, 31 were LMICs and LICs, most of which belonged to Western and Central Africa (n = 16) and Eastern and Southern Africa (n = 10). Figure 1 shows^24^ the distribution of COVID-19 vaccines to every country as a percentage of the country’s total population by February 2022.^25,26^ Beyond COVID-19 are health security threats like, One Health including Antimicrobial Resistance^27^ and zoonosis,^28^ which are exacerbated through climate change and deforestation,^29^ thus posing long-term risks to global stability through a need for coordinated solutions in diplomacy.

###  Development and economic implications

 There is an intrinsic link between health and development,^30^ whereby health outcomes can often be a determinant of economic growth.^31^ As per the WHO, such health investments have a multiplier effect, enabling workforce productivity and reducing poverty, although the economic impact of a health crisis often derails developments.^32^ The Ebola outbreak from 2014 to 2016 in West Africa cost the region around $2.8 billion to $32.6 billion in GDP losses,^33^ emphasizing a need for robust health diplomacy to mitigate such disruptions.

 Integration efforts of health resilience into global economic strategies have been through initiatives such as the *G20’s Joint Finance and Health Task Force*.^34^ This initiative emphasizes financing mechanisms for pandemic preparedness and response and, in doing so, is ensuring that future outbreaks will not disproportionately affect vulnerable economies. Bilateral and multilateral partnerships have emerged as critical vehicles for addressing the dual objectives of economic recovery and health systems strengthening. For example, initiatives such as the COVAX Facility show how multilateral work can increase vaccine equity with international cooperation.^35^

###  Bilateral and multilateral relations: Through health diplomacy

 Bilateral and multilateral relations are key pillars of health diplomacy, facilitating coordinated responses to shared challenges. Bilateral health initiatives often focus on direct aid and vaccine donations, such as providing over 1 billion COVID-19 vaccine doses by the *COVAX*, The United States to LMICs and low-income countries (LICs).^24^ These efforts address the immediate health needs and strengthen diplomatic ties and regional stability. On the other hand, multilateral platforms, such as the WHO, G20, and the United Nations, provide for collective decision-making, mobilization of resources, and harmonization of health policies with the UN sustainable development goals. Recent G20 discussions have underscored multilateralism as necessary for building resilient health systems.^36^ Initiatives such as the Global Health and Finance Board have tried to relate financial priorities to health outcomes and advocated sustainable funding sources for global health emergencies.^37^ Multilateral work underscores the need to draw resources and expertise together on complex health and development agendas.

###  Literature gaps and the need for a comprehensive analysis

 Despite the growing relevance of health diplomacy, the academic literature remains fragmented. Existing studies often focus narrowly on specific aspects, such as pandemic response or bilateral vaccine agreements, leaving broader questions unaddressed. For example, few analyses explore the intersection of health security with trade policies or the impact of multilateral platforms like the G20 on vaccine equity and strengthening global health systems. Furthermore, bibliometric studies mapping the evolution of health diplomacy research and its policy impacts are scarce, limiting our ability to understand trends, identify gaps, and guide future inquiry. Therefore, this study aims to bridge the gaps outlined by addressing them with a bibliometric analysis of GHD, particularly in the interest of health security, development, and international cooperation to 1) Map the research landscape: Trends, key contributors, and geographic patterns in health and vaccine diplomacy research. 2) Explore the thematic areas by investigating the connections between health diplomacy, economic development, bilateral and multilateral relations, and health security. 3) Evaluate policy implications by examining the role of health diplomacy in addressing global health challenges for equitable health outcomes and 4) Inform future policy decisions by offering evidence-based understanding to direct global health policy, boost multilateral collaborations, and strengthen health systems.

## Methods

###  Data source and search strategy

 This study utilized SCOPUS, a premier database for peer-reviewed literature in all fields, to analyse the academic literature on GHD. The search was performed with a focused strategy using the keyword “global health diplomacy” in the TITLE-ABS-KEY field. This resulted in only articles with the phrase “global health diplomacy” in their title, abstract, or keywords in the dataset. The search produced 242 results, including articles in journals across a wide range of disciplines, from global health to public policy and international relations.

 No restrictions were placed on the date of publication or the geographic location of the studies, which enables a comprehensive longitudinal analysis of the evolution of research in this field. Articles not relevant to the topic, including those that focus on peripheral or tangential aspects, were excluded during data cleaning to ensure the specificity and relevance of the dataset.

###  Data extraction and cleaning

 After the search result retrieval, metadata such as title, abstract, keywords, year of publication, the authors’ affiliations, journal titles, and their respective citation counts were exported to be analyzed. The dataset underwent rigorous cleaning by removing duplicates, non-English articles, and studies irrelevant to the core issues of GHD. The cleaned dataset of 239 articles was used to conduct bibliometric and thematic analyses. Three studies were excluded as they were retracted or corrected articles of published articles.

###  Analytical tools and processes

 The study employed a two-tiered approach: bibliometric analysis and thematic analysis, leveraging Biblioshiny^38^ and Microsoft Excel for detailed insights. We carried out the study using Biblioshiny as a web interface for a bibliometric R-based package. Data analyses included determining publication trends, citation metrics, and authorship patterns. Critical indicators include publication output every year, the most frequently cited articles in the network, and geospatial research distribution for the network.

 Co-authorship network analysis and keyword co-occurrence networks were made to determine clusters of collaborators and thematic features in the data set.

 To complement bibliometric insights with qualitative depth, the dataset was exported to Microsoft Excel for thematic analysis. Articles were manually analysed based on recurring themes, including health security, vaccine diplomacy, international cooperation, economic development, and trade policy. Abstracts and keywords were reviewed to look for underlying research priorities, gaps, and emerging areas of interest. Thematic analysis contextualized the findings from the bibliometrics and highlighted the breadth and depth of research in the field.

## Results

Table 1 indicates that from 2007 to 2024, research on GHD comprises 239 documents sourced from 135 publications. There are 691 contributing authors, with 43.1% engaging in international co-authorship. The average number of co-authors per document is 3.98, and 49 are single-authored. The documents reflect an annual growth rate of 7.65%, with an average citation count of 13.67 and a document average age of 6.51 years. There were 488 unique author keywords found in the dataset.

**Table 1:** Bibliometric summary of global health diplomacy research (2007–2024)

**Metric**	**Value**
Timespan	2007-2024
Sources	135
Documents	239
Annual growth rate	7.65%
Authors	691
Authors of single-authored documents	49
International co-authors	43.1%
Co-authors per document	3.98
Author's keywords	488
Document average age	6.51
Average citations per document	13.67

Figure S1 (Supplementary file 1) displays the annual scientific production of articles related to GHD from 2007 to 2024. The production has been fluctuating, but with clear peaks in 2013 and 2022, indicating a rise in research activity at these points. An overall upward trend in production indicates an increasing interest in the field.

Figure S2 (Supplementary file 1) gives an overview of average citations per year for articles on GHD, 2007-2024. The average citations per year peaked in 2008 and 2010, then decreased sharply and stabilized at lower levels thereafter. Recent years are trending upward again before a sharp decline in 2023, indicating fluctuating academic influence over time.

**Figure 2:**
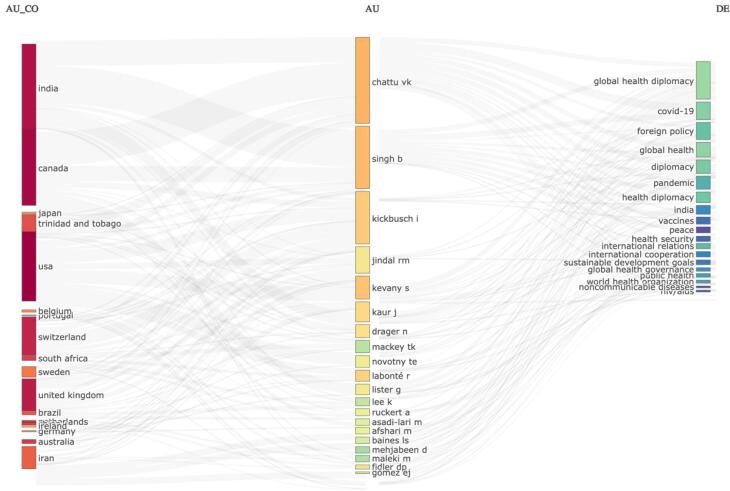
Three-field plot of countries, authors, and keywords (source: prepared by the authors using Biblioshiny)

Figure 2 illustrates the links between participating countries (left), core authors (middle), and frequently used keywords (right) in GHD research. India, the USA, and Canada are some of the key contributors; notable authors such as Chattu VK, Singh B, and Kickbusch I are linked to central themes like “global health diplomacy,” “COVID-19,” and “health security.” The figure demonstrates the collaborative nature of research across countries and the alignment of author focus areas with key global health challenges.

**Figure 3:**
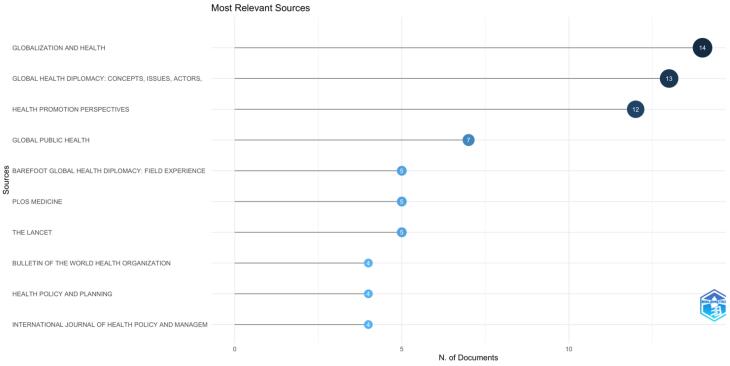
Most relevant sources for global health diplomacy research (source: prepared by the authors using Biblioshiny)

Figure 3 highlights the most relevant journals (sources) publishing research on GHD. The journal *Globalization and Health* leads with 14 documents, followed by *Global Health Diplomacy: Concepts, Issues, Actors* with 13 documents and *Health Promotion Perspectives* with 12. Other leading sources include *Global Public Health* (7), *Barefoot Global Health Diplomacy* (5), and journals like *The Lancet* and *PLOS Medicine,* contributing five documents each.

Figure S3 (Supplementary file 1) depicts the H-index of the most impactful sources in GHD research. Journals such as *Global Public Health*, *Globalization and Health*, and *Health Promotion Perspectives* each have an H-index of 6, indicating significant local impact. Other sources, including *Global Health Diplomacy: Concepts, Issues, Actors* and *PLOS Medicine*, follow with an H-index of 5. Journals such as the *Bulletin of the World Health Organization* and *BMC Public Health* have slightly lower H-indices, demonstrating varied levels of influence within the field.

Figure S4 (Supplementary file 1) displays the cumulative production of key sources contributing to GHD research from 2007 to 2024. *Globalization and Health* and *Global Public Health* show continuous growth in their publications over the years, showing sustained contributions to the field. Other sources, such as *Health Promotion Perspectives* and *Global Health Diplomacy: Concepts, Issues, Actors*, demonstrate a sharp increase in production after 2010, with continued additions in recent years. The figure indicates varying growth rates among sources, highlighting their evolving roles in shaping the research landscape.

**Figure 4:**
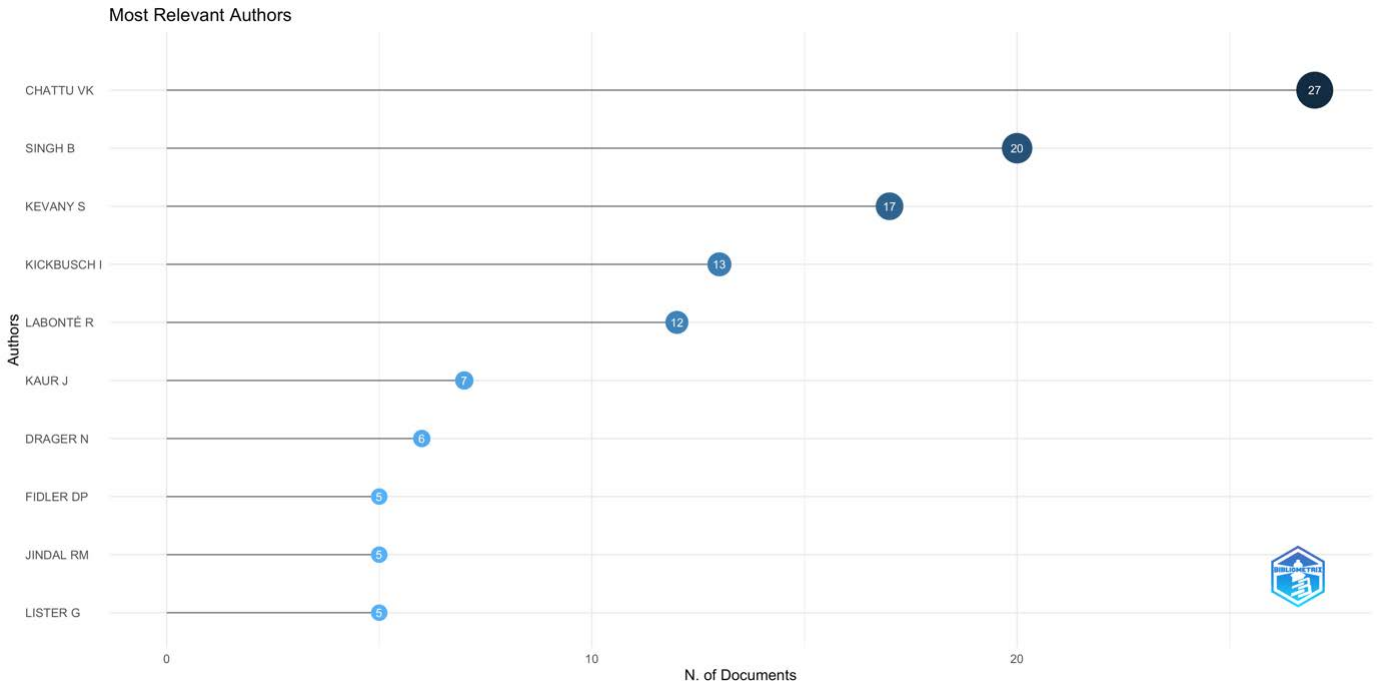
Most relevant authors in global health diplomacy research (source: prepared by the authors using Biblioshiny)

Figure 4 shows the most relevant authors based on their contributions to GHD research. Chattu VK is the leading author with 27 documents, followed by Singh B with 20 and Kevany S with 17. Other significant contributors include Kickbusch I (13 documents) and Labonté R (12 documents). Authors like Kaur J, Drager N, Fidler DP, Jindal RM, and Lister G have also made notable contributions, with 5 to 7 documents each. This figure highlights the prominent researchers shaping the field.

Figure S5 (Supplementary file 1) highlights the most relevant institutional affiliations contributing to GHD research. The University of Toronto leads with 36 articles, followed by the University of California with 29 and the University of Ottawa with 22. Other significant contributors include the Central University of Punjab (17 articles) and the Graduate Institute of International and Development Studies (14 articles). Institutions such as McGill University and Iran University of Medical Sciences have contributed eight (8) articles each, reflecting diverse geographic and academic engagement in the field.

Figure S6 (Supplementary file 1) depicts the overall growth in article production on GHD by country over time. The USA leads with the highest publications, followed by Canada and India, which show significant and consistent growth. The United Kingdom and Switzerland also contribute steadily but at a comparatively lower rate. The figure highlights the geographical distribution of research contributions and the dominant role of North American and South Asian countries in advancing the field.

Figure S7 (Supplementary file 1) highlights the most globally cited documents in GHD research. The article by Labonté and Gagnon^39^ published in *Globalization and Health* leads with 168 citations, followed by Kickbusch et al^40^ in *Bulletin of the World Health Organization* with 150 citations. Another highly influential work is Labonté et al^41^ in *Annual Review of Public Health*, which has been cited 129 times, and Fidle ^42^ in *PLOS Medicine* with 128 citations. Other notable contributions include Fidler^43^ in *Emerging Infectious Diseases* (124 citations), Feldbaum and Michaud^44^ in *PLOS Medicine* (120 citations), and Katz et al^4^ in *Milbank Quarterly* (105 citations). Additionally, Adams et al^45^ in *Medical Anthropology Cross Cultural Studies of Health and Illness* and Kaufmann^46^ in *Health Affairs* each have 100 citations, while Lee et al^47^ in *PLOS Medicine* has been cited 66 times. These documents collectively represent the foundational works that have significantly shaped the discourse in GHD.

Figure S8 (Supplementary file 1) visualizes the trend topics in GHD research over time, highlighting the frequency of terms used in publications. Recent years show a significant increase in terms like “coronavirus disease 2019,” “COVID-19,” “pandemic,” and “SARS-CoV-2,” reflecting the field’s response to global health emergencies. Other frequently occurring terms include “global health,” “diplomacy,” “public health,” “health policy,” and “international cooperation,” which have remained consistent themes across the timeline. Emerging topics like “capacity building,” “sustainable development,” and “leadership” indicate a broadening scope of research priorities. This figure demonstrates the evolving focus areas and growing diversification within the field.

**Figure 5:**
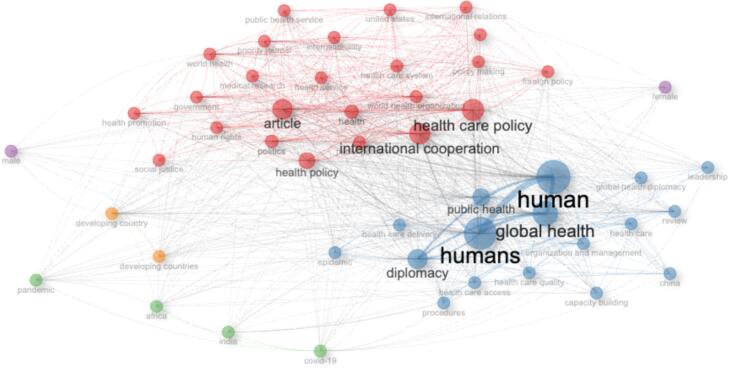
Co-occurrence network of keywords (source: prepared by the authors using Biblioshiny)

Figure 5 illustrates the co-occurrence network of keywords in GHD research, showing clusters of frequently associated terms. Central themes include “human,” “global health,” “diplomacy,” “public health,” and “health care policy,” which are interconnected with broader topics like “international cooperation,” “health policy,” and “social justice.” Additional clusters highlight specific areas such as “pandemic,” “capacity building,” and “developing countries,” reflecting the diversity and interrelation of research priorities.

**Figure 6:**
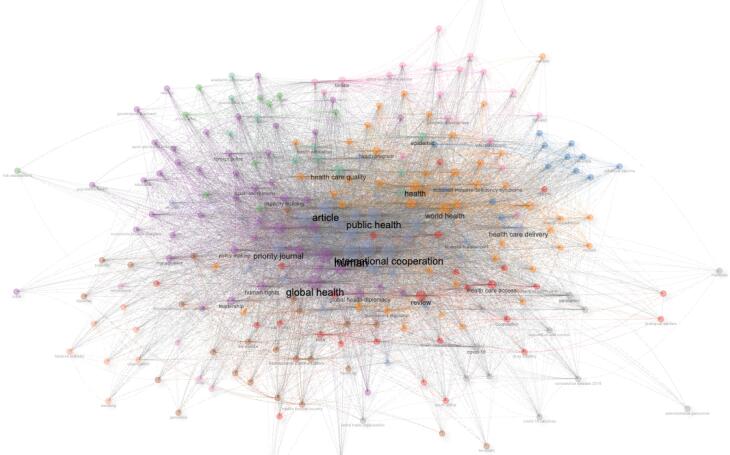
Thematic keyword co-occurrence network (source: prepared by the authors using Biblioshiny)

Figure 6 presents a thematic keyword co-occurrence network, emphasizing the interconnectedness of central themes in GHD research. Prominent keywords like “public health,” “global health,” “international cooperation,” and “human” occupy central positions, highlighting their foundational role in the discourse. The network reveals strong associations with terms such as “health care delivery,” “pandemic,” “policymaking,” and “capacity building,” indicating critical intersections of research focus areas. Peripheral terms like “tobacco industry,” “negotiations,” and “smoking” demonstrate niche but relevant aspects within the broader research context.

## Discussion

 This study delivers a detailed bibliometric analysis of GHD between 2007 and 2024 and presents crucial trends in authorship, institutional collaboration, thematic focus, and policy implications. A total of 239 articles from the SCOPUS-indexed sources were used, with a yearly growth rate of 7.65%. Thematic development highlighted a huge emphasis on infectious diseases, vaccine diplomacy, and global health governance during the COVID-19 pandemic. The dominance of authors such as Chattu VK and institutions such as the University of Toronto signifies high-income countries’ leading position in GHD research. Similar to findings by Ruckert et al^48^ and Labonté & Gagnon,^39^ this study reaffirms that research output in GHD is predominantly led by North American and European countries, with the United States and Canada being top contributors. The dominance of such institutions as the University of Toronto and the University of California supports earlier observations made by Wang et al^49^ regarding the concentration of global health research in high-income settings. However, this study also pointed out emerging contributions from LMICs such as India and South Africa, a reflection of the growing interest of these regions in the use of health diplomacy to serve both domestic and international agendas.

 The themes identified here resonate closely with the overall health issues that have been highlighted in the literature. For instance, Kickbusch et al^40^ highlighted an increased role of health in foreign policy and governance; the same is echoed here through the prominence of keywords such as “global health governance” and “international cooperation.”. Similarly, a key area that Feldbaum and Michaud^44^ pointed out was that of vaccine diplomacy, yet this has become more current in the context of COVID-19. However, our search also points to a lack of recent research on noncommunicable diseases (NCDs) and health-climate relations despite increasing advocacy on these topics, as reported by Afshari et al.^50^

 This study reveals critical emerging themes and gaps in GHD for which future research can be done and policies can be implemented (Table 2), showing the evolving nature of this interdisciplinary field. The thematic approach shows that the dominance of infectious diseases during global health crises, like the COVID-19 pandemic, has shaped the research trajectory. Vaccine diplomacy, global health governance, and international cooperation are recurring priorities, emphasizing health and foreign policy intersection. Still, several underexplored areas persist, pointing to substantial gaps in existing literature.

**Table 2:** Policy and research recommendations based on study findings

**Observation**	**Policy recommendation**	**Research recommendation**
Uneven regional representation	Support LMICs-led collaborations	Conduct comparative regional analyses
Focus on infectious diseases	Promote the inclusion of NCD-related themes	Study interactions of climate change and health
Dominance of high-income countries	Facilitate equitable funding mechanisms	Expand co-authorship with LMIC researchers
Emergence of vaccine diplomacy	Develop policies for equitable access	Investigate geopolitical implications

## Strengths and limitations

 The strengths of this study are that it employed a comprehensive bibliometric methodology in systematically mapping the landscape of GHD research. However, the study’s limitations include relying on SCOPUS as the sole database, which might exclude relevant studies indexed elsewhere. The single-keyword approach might also have omitted studies using alternative terminologies, a limitation also acknowledged by Wang et al.^49^ Another methodological pitfall in conducting this study was considering published articles only in English language there by omitting articles published in other languages.

## Conclusion

 This study contributes to a nuanced understanding of GHD by identifying key trends, thematic gaps, and policy implications. While progress has been made, significant efforts are required to address the regional and thematic disparities in this field. Future research should prioritize interdisciplinary approaches and equitable representation to enhance the global impact of health diplomacy.

## Competing Interests

 Vijay Kumar Chattu is an editorial board member of *Health Promotion Perspectives*. Other authors declare no competing interests.

## Ethical Approval

 Not applicable.

## Supplementary Files


Supplementary file 1 contains Figures S1-S8.
