# Supplementary material for: Bridging international borders through global health diplomacy: A comprehensive bibliometric analysis of the state of play and leads for advancing this domain
Source: Health Promot Perspect. 2025 Nov 4;15(3):252–60. doi: 10.34172/hpp.025.44650 (PMC12680524; doi:10.34172/hpp.025.44650)
Supplement: Supplementary file 1 — contains Figures S1-S8. [file hpp-15-252-s001.pdf]

Mittal et al, **Health Promotion Perspectives.** 2025;15(3):252-S1
doi: 10.34172/hpp.025.44650
https://hpp.tbzmed.ac.ir

Supplementary file 1

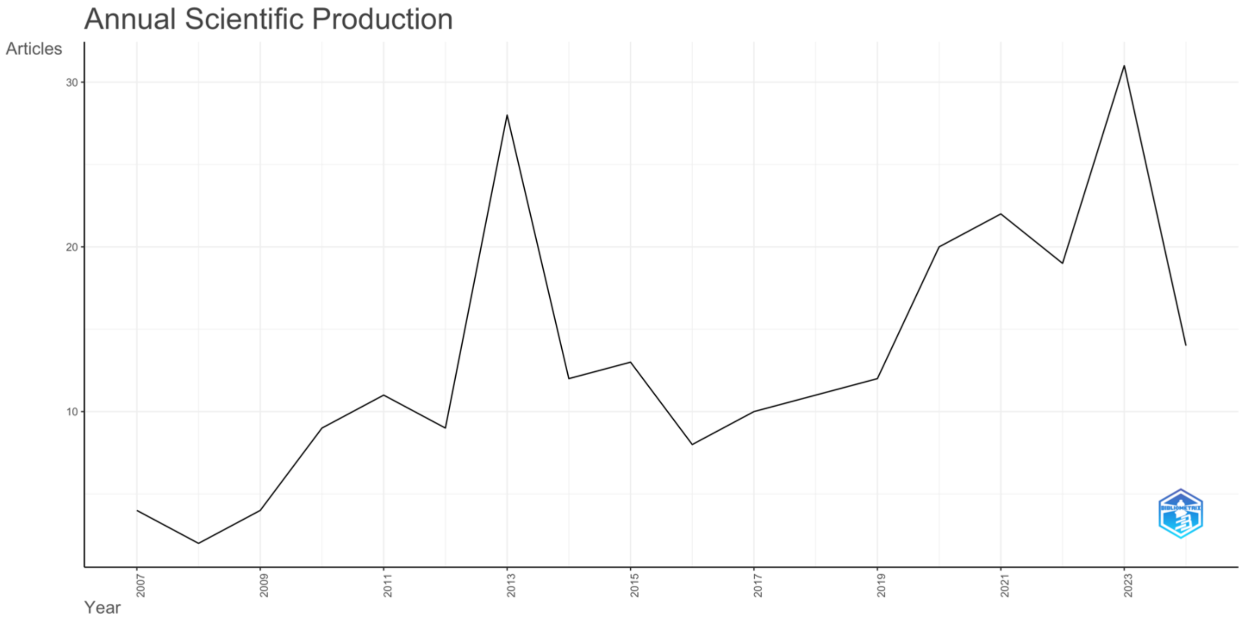

Figure S1 : Annual Scientific Production (2007–2024) (**source**: prepared by the authors using Biblioshiny)

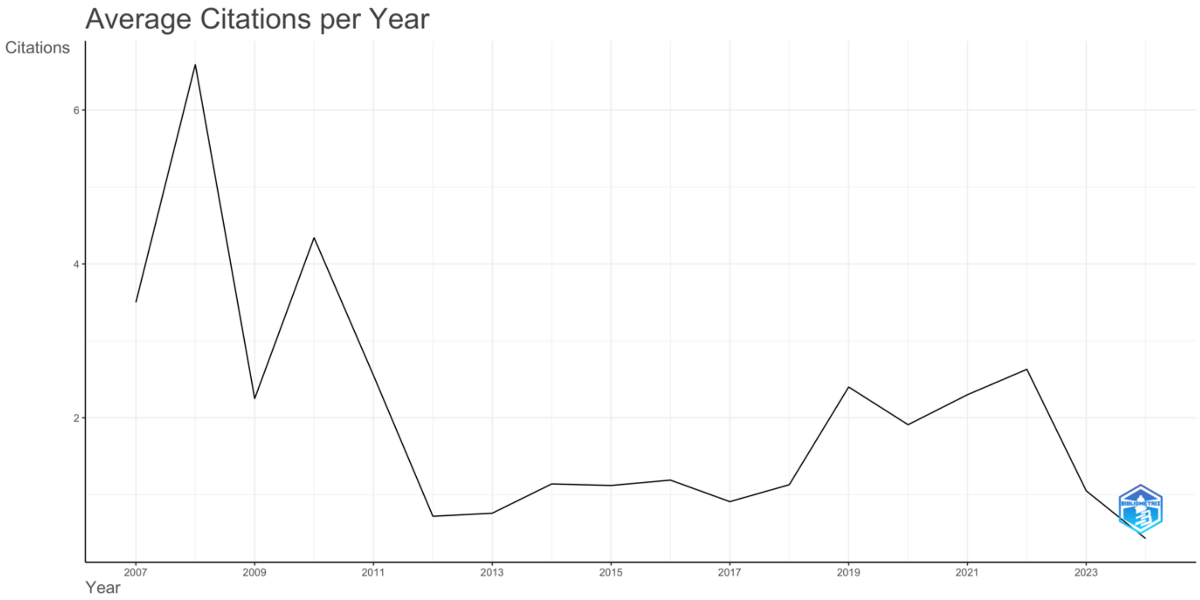

Figure S2 **:** Average Citations per Year (2007–2024) (**source**: prepared by the authors using Biblioshiny)

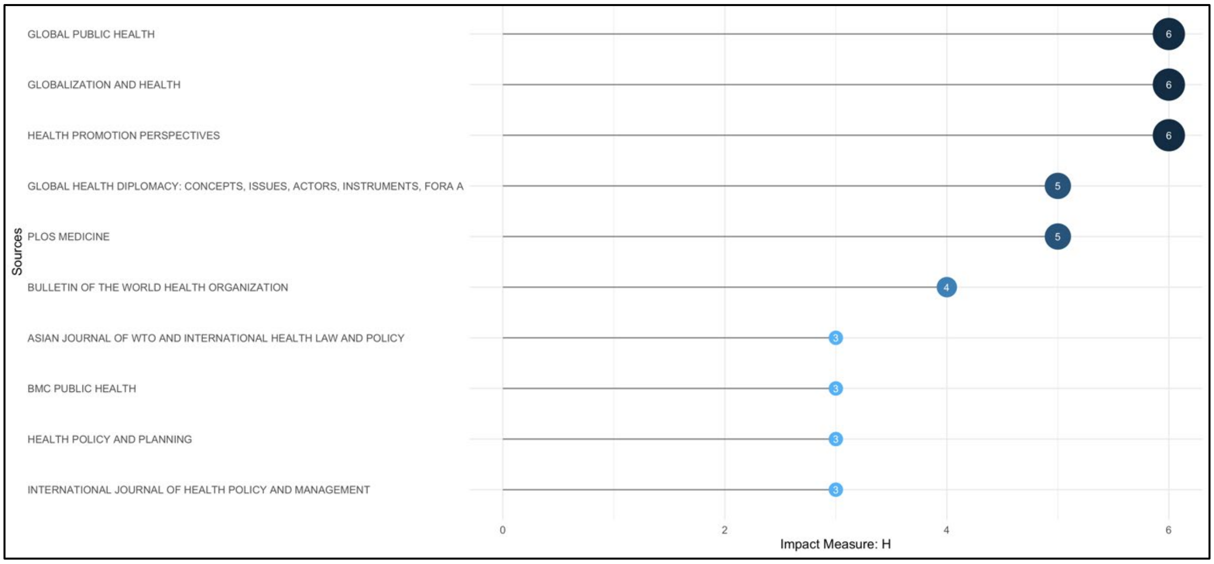

Figure S3**:** Sources' Impact by H-Index

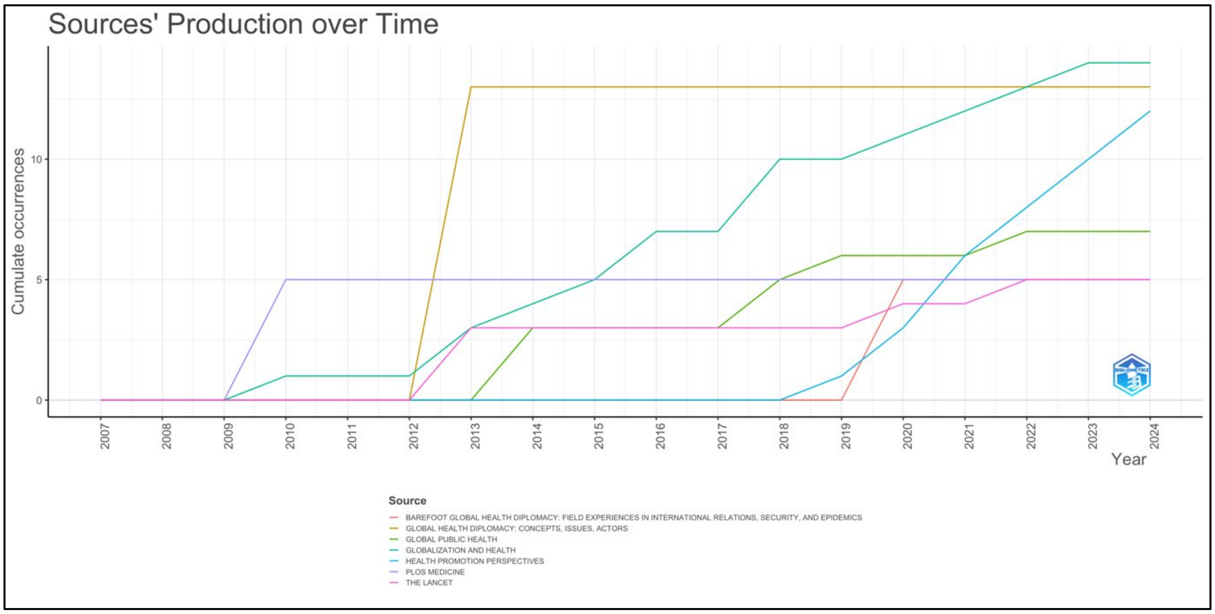

Figure S4**:** Sources' Production Over Time (**source**: prepared by the authors using Biblioshiny)

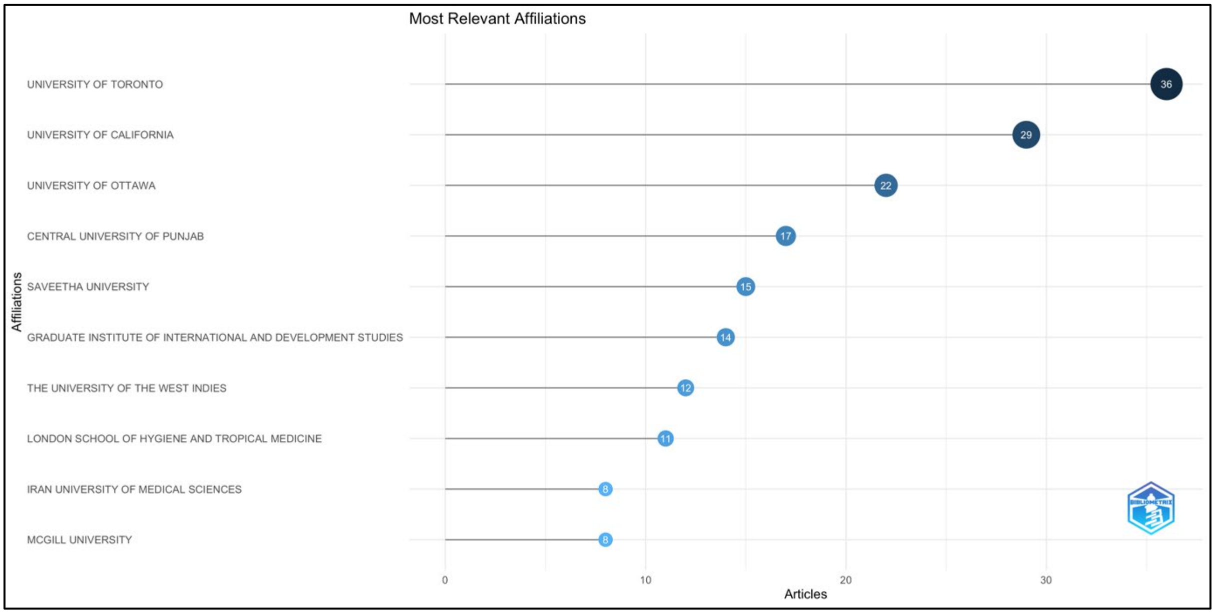

Figure S5**:** Most Relevant Affiliations in Global Health Diplomacy Research (**source**: prepared by the authors using Biblioshiny)

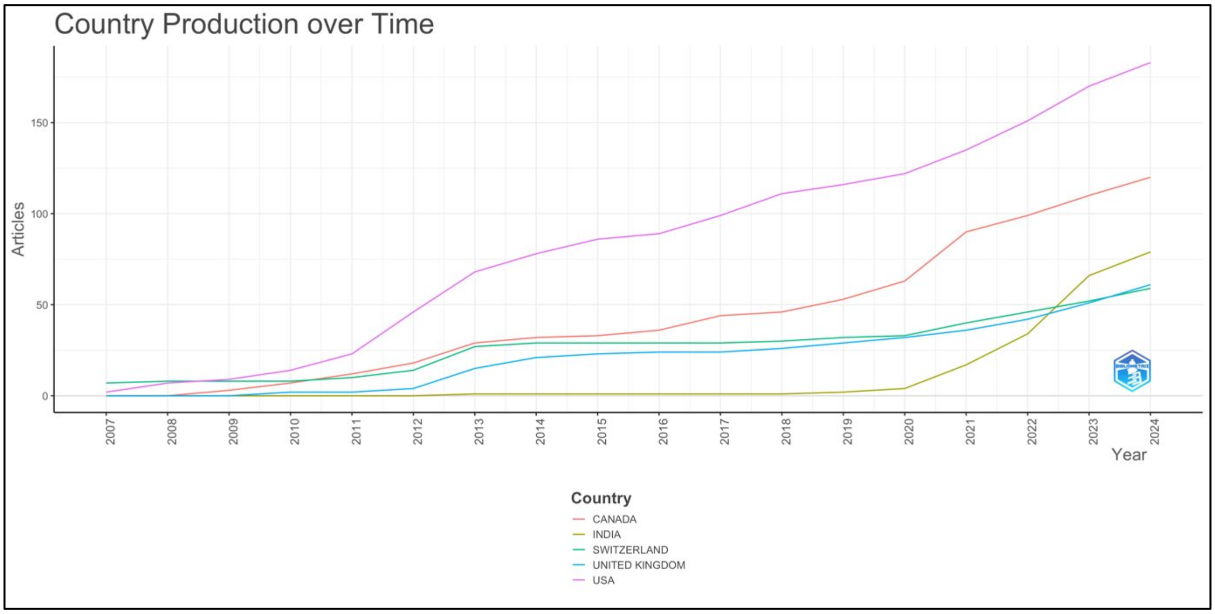

Figure S6**:** Country Production Over Time (2007–2024) (**source**: prepared by the authors using Biblioshiny)

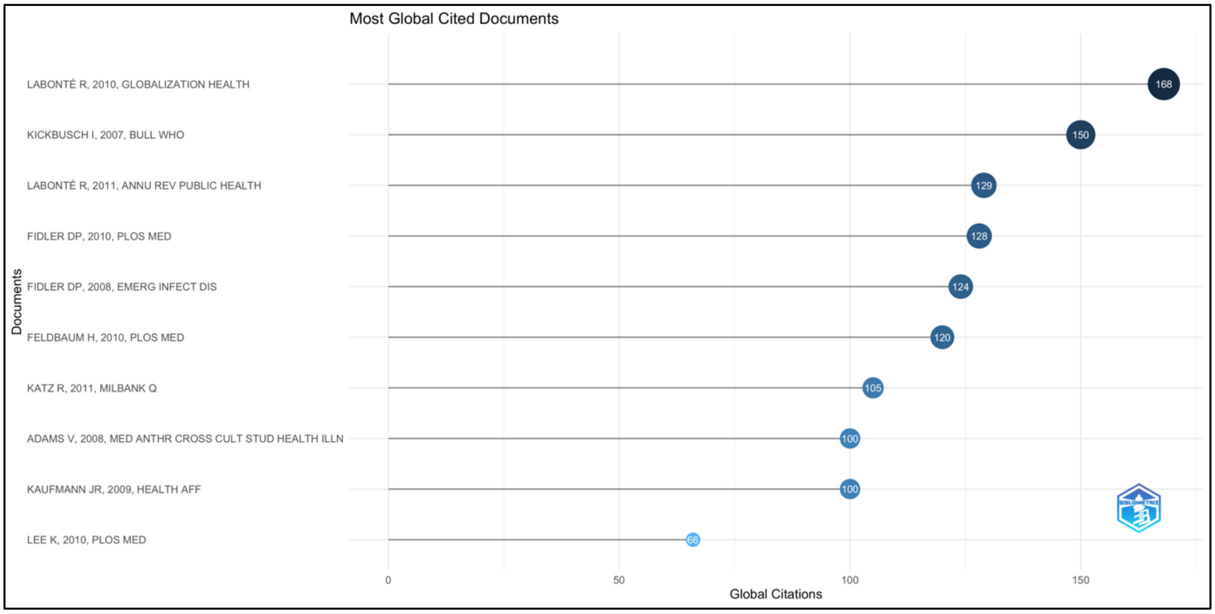

Figure S7**:** Most Globally Cited Documents in Global Health Diplomacy Research (**source**: prepared by the authors using Biblioshiny)

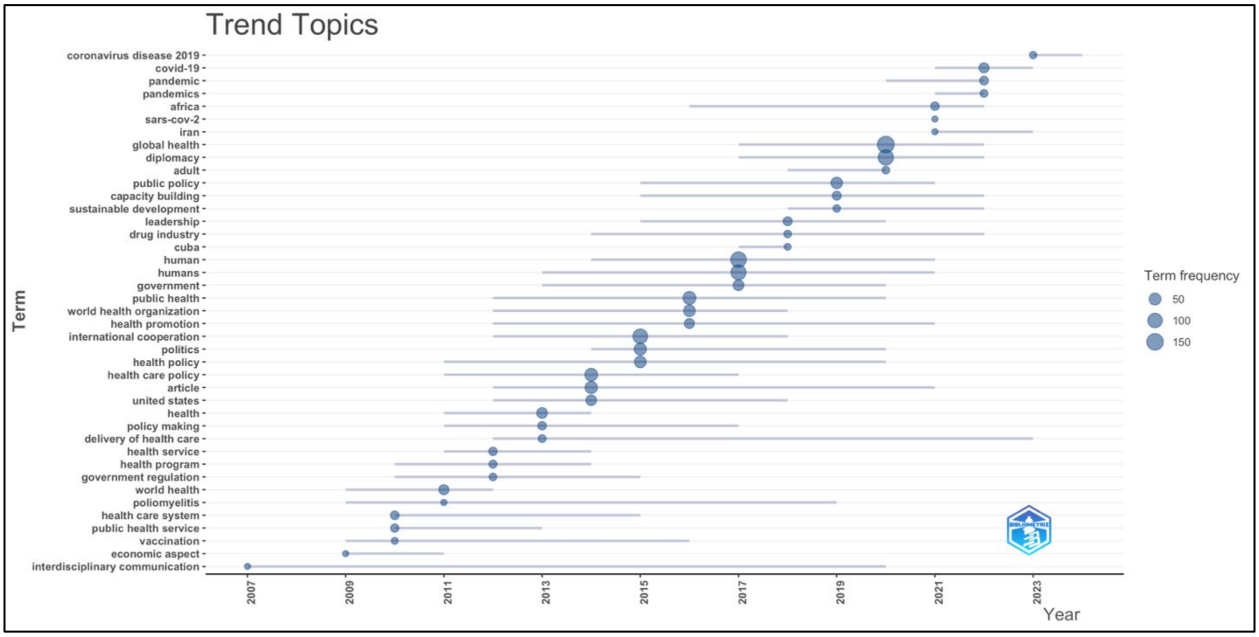

Figure S8**:** Trend Topics in Global Health Diplomacy Research (**source**: prepared by the authors using Biblioshiny)
